# Neural specialization to human faces at the age of 7 months

**DOI:** 10.1038/s41598-022-16691-5

**Published:** 2022-07-21

**Authors:** Santeri Yrttiaho, Anneli Kylliäinen, Tiina Parviainen, Mikko J. Peltola

**Affiliations:** 1 Human Information Processing Laboratory, Faculty of Social Sciences, Tampere University, Tampere, Finland; 2 Tampere Institute for Advanced Study, Tampere University, Tampere, Finland; 3 Department of Psychology, Center for Interdisciplinary Brain Research (CIBR), University of Jyväskylä, Jyväskylä, Finland

**Keywords:** Neuroscience, Psychology

## Abstract

Sensitivity to human faces has been suggested to be an early emerging capacity that promotes social interaction. However, the developmental processes that lead to cortical specialization to faces has remained unclear. The current study investigated both cortical sensitivity and categorical specificity through event-related potentials (ERPs) previously implicated in face processing in 7-month-old infants (N290) and adults (N170). Using a category-specific repetition/adaptation paradigm, cortical specificity to human faces, or control stimuli (cat faces), was operationalized as changes in ERP amplitude between conditions where a face probe was alternated with categorically similar or dissimilar adaptors. In adults, increased N170 for human vs. cat faces and category-specific release from adaptation for face probes alternated with cat adaptors was found. In infants, a larger N290 was found for cat vs. human probes. Category-specific repetition effects were also found in infant N290 and the P1-N290 peak-to-peak response where latter indicated category-specific release from adaptation for human face probes resembling that found in adults. The results suggest cortical specificity to human faces during the first year of life. Encoding of unfamiliar cat stimuli might explain N290 amplification found in infants.

## Introduction

Early capacities^1,2^ and biases^3,4^ for face processing as well as their importance for further development^1,3,5,6^ have been well-documented in both behavioral^7^ and electrophysiological^8^ measures. The cortical basis of face processing in infants has predominately been studied using event-related potentials (ERP), where face-sensitivity has been found during the first year of life in amplitude of the occipitotemporal N290 and P400 responses^8–11^ already resembling the adult N170 response^12^ to a considerable degree. However, the organization and development of the neural resources activated by faces have remained unclear^6,13,14^. Importantly, early sensitivity to faces found in cortical responses as such does not constitute as evidence for specific tuning to faces in the involved cortical regions as these might be activated by other object categories as well^14,15^. For understanding the developmental processes leading to mature functioning of the face processing network, accurate mapping of neural representations of conspecific faces in infancy are thus needed.

Accounts of the developmental fine-tuning of face-specific brain regions range from primarily biologically hard-wired to more experience-dependent accounts^14^. According to the maturational view, domain-specific face processing found in the adult brain (e.g., the fusiform face area) develops due to intrinsic genetic factors that induce local neuroanatomic changes^14^. The concept of *interactive specialization* (IS^16,17^), contrasts the maturational view and suggests that face processing networks emerge and specialize through interactions between initially unspecialized brain regions and the environment. According to the IS view, the neural resources sensitive to faces are initially broadly tuned and become more narrowly tuned to faces (as opposed to other objects) partly analogously to the developmental fine-tuning of receptive fields on a cellular level^18^. While early sensitivity to faces may be compatible with both IS and maturational models, the IS model suggests that the neural assemblies responsive to faces would be more broadly tuned to various closely related stimuli such as non-human faces^14^ or inverted faces^19^ in infants than in adults. Thus, the IS model predicts categorical specificity to faces in adults but not in infants. In contrast, according to the maturational view, the early cortical sensitivity to faces^8–11^ suggests that the involved neural regions would also be selectively tuned to human faces as opposed to closely related stimuli.

The current study investigated the predictions of the maturational and the IS model for face processing early in life by studying sensitivity and categorical specificity of infant brain responses to human face and to matched control stimuli. We used a category-specific repetition paradigm^20–22^, where ERPs are elicited by *probe* stimuli that are preceded by specific *adaptors* (or *primes*). Typically, a decreased response amplitude, adaptation, is found when categorically similar probe and adaptor stimuli are repeated in comparison to conditions with categorically dissimilar probes and adaptors^23,24^. Conversely, a relative increase in ERP amplitude, or release from adaptation, is found when the repetition of the probe stimuli is paired with categorically dissimilar adaptors. These stimulus-specific effects have been attributed to reflect selective activity in distinct neural resources as elicited by stimuli across different modalities such as intensity, frequency^25^ and speech-related features^26^ of auditory stimuli, specific types of visual information from orientation tuning to color, motion, object identity and faces^23^, as well as language and semantic categories^27^ in infants, children, and adults^21^. In contrast, the absence of stimulus-specific effects across variable probe and adaptor stimulus categories could be taken to suggest considerable overlap between broadly tuned neural resources activated across the investigated stimulus features^23^. It is of note that an effect in the opposite direction to adaptation has been found, where the repetition of a stimulus category leads to response amplification (rather than suppression) through category-specific priming^21^. The direction of the repetition effect has been systematically associated with factors such as stimulus familiarity and the number of stimulus repetitions^21^.

In the present study, cortical face-sensitivity was defined as increased amplitude of the infant ERPs in response to human faces as opposed to control stimuli. Categorical specificity of ERP responses to faces was, in turn, investigated through stimulus-specific repetition effects^21,25,26^. In our experiment, we included four conditions where human face (*F*) and control stimuli (cat faces, *C*) served both as probe and as adaptor stimuli in following combinations: human face probe alternated with human adaptor (*FF*), human face probe with cat adaptor (*FC*), cat probe with cat adaptor (*CC*), and cat probe with human face adaptor (*CF*). Category-specific release from adaptation, as indicative of category-specific tuning, was operationalized as increased ERP amplitude elicited by face probes alternated with categorically dissimilar (cat) adaptors in contrast to categorically similar face adaptors (*FC* > *FF* or *CF* > *CC*). While the maturational model would predict both face sensitivity (*FF* > *CC*) and category specificity (*FC* > *FF*) in the infant ERPs, the IS model would predict sensitivity (*FF* > *CC*) without categorical specificity (*FC* = *FF*).

The current study investigated neural representation of faces in infants at 7 months of age. Biases in the neural level to face vs. various control stimuli have been indicated in infant participants already at 3 months of age as indicated in the N290 response^9,10^. However, both neural and behavioral responses to faces undergo changes during the first year of life. Facial individuation and specialization to conspecific faces has been shown to take place between 6 and 9 months^28^ paralleled by findings from the N290 response^29^. Other research has indicated basic level categorization by 6–7 months of age to variable object categories, although at delayed latencies corresponding to the Nc response in ERP studies^30^. In this study, we focused on infants at 7 months of age as the abilities to recognize facial cues indicate significant development between 5 and 7 months of age^11,31^ which might lead to categorical face representation already by 7 months of age. We focused on activity elicited around 290 ms after stimulus onset in the infant occipitotemporal cortex (N290) which has been previously implicated in face processing^8–11^. For validation of the applied protocol, we also collected N170 (elicited around 170 ms after stimulus onset) response from adult participants which, based on previous studies, was expected to be both sensitive and category-specific to face vs. control stimuli^32–34^ thus indicating specific tuning to faces.

## Methods

### Participants

Infant participants were recruited from Tampere and surrounding areas. Contact information for families with infants around 7 months of age during data collection (October 16th–December 7th, 2020) were drawn from the Finnish Digital and Population Data Services Agency. Out of 521 contacted families, 63 volunteered and 56 brought their infant child to a study visit. Data from altogether 49 (87.5%) infants [19 female, age = 30.5 (0.5) weeks] were qualified into the final analyses. As background data we collected parent-reported temperament traits of infant participants using the IBQ-R short form^35^ (see Supplementary Material for further details).

Altogether 25 adults participated in the ERP experiment. The sample was recruited from psychology undergraduates and their social networks. Participants reported no visual or neurological impairments. After EEG preprocessing and artefact detection, data from 21 [11 female, age = 26.7 (7.7) years] participants were qualified for final analyses. Data from the four rejected participants included excessive impedances in several electrode channels (> 10% of all channels).

The experimental protocol and the methods were in accordance with the ethical principles of research with human participants and ethical review in the human sciences in Finland and with the institutional guidelines and regulations provided by the Ethics Committee of the Tampere Region. The Ethics Committee of the Tampere Region approved the current study, its methodology, and implementation (statement 29/2020). Prior to participation, written informed consent was given by the participants or parent of the infant participants before the start of the study.

### Stimuli

**Figure 1:**
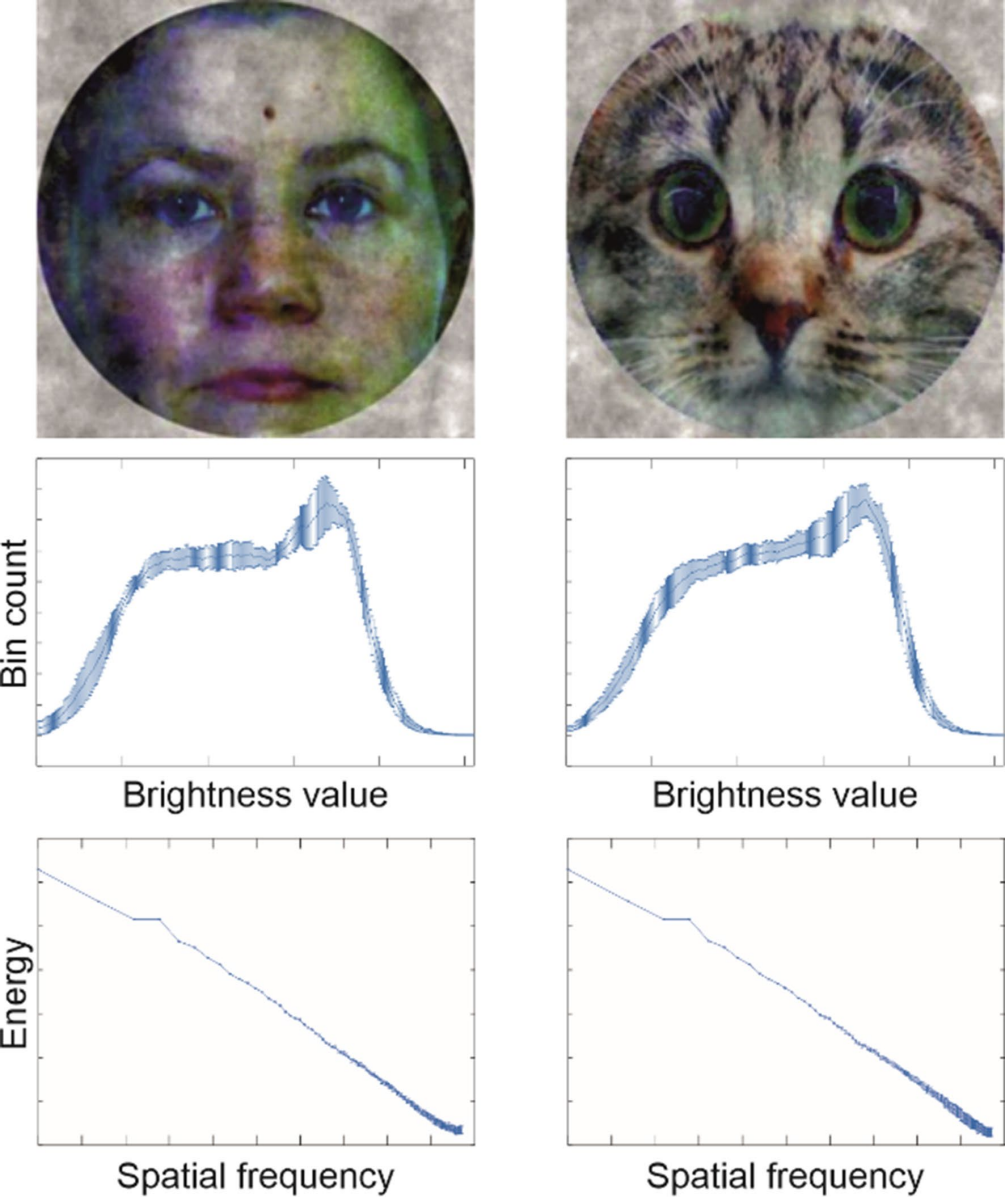
Human and cat face stimuli. Human face stimuli consisted of photographs of seven female individuals and were modified from images included in the *Utrecht ECVP face database.* The cat stimuli consisted of seven exemplars of short-haired cats including and resembling the depicted stimulus example. Low-level stimulus features were matched across stimuli *using manipulations of the luminance histograms and spatial frequency spectra of the images.* Top panel: example stimuli from human (left) and cat (right) categories. Middle panel: Luminance histograms from human (left) and cat (right) stimuli indicate brightness values (0–255) on the *x*-axis and count of pixels at each value on the *y*-axis. Bottom panel: Spatial frequency spectra of human (left) and cat (right) faces. The x-axis indicates frequency as cycles/image. The *y*-﻿axis shows energy at frequency. Line-plots indicate the mean value across stimuli and error-bars indicate standard deviation across exemplars (for both histograms and spectra alike). The human face photograph depicted above, resembling those used in the current study, is included here with permission from the model. The cat photograph is a derivative of “European_shorthair_TUROK_cat_show_Turku_2010-03-27.JPG” by Heikki Siltala licensed under CC BY 3.0.

The human face stimuli were adapted from the Psychological Image Collection at Stirling^36^. Cat faces, which were used as control stimuli, were gathered from photographic pictures available over the internet (public domain or licensed under CC BY 3.0). To avoid excessive within-category variability, the human face pictures selected for stimuli consisted of female faces with neutral or mild positive facial expressions. The cat stimuli were chosen to consist of a relatively homogenous exemplars from a short hair breed. Altogether 7 different human and 7 different cat face pictures were included in the current set of stimuli. Examples of human and cat face stimuli are shown in Fig. 1.

The background of all pictures, surrounding human or cat faces, were replaced by a uniform white background. Cat faces were then stretched vertically to align the level of the eyes and the mouth to match those of human faces. The pictures were then cropped with a circular frame with a diameter matching the 256 by 256-pixel area of the picture. The color values of the images were modified by using histogram equalization across RGB levels with GIMP software^37^ and by then equalizing the histograms of pixel intensities (range 0–255, mean: 121) across all images with the Shine_color toolbox^38^. Mean RGB intensities between human and cat images indicated negligible color differences^39,40^ between the stimulus categories (0%, 1%, and 1% for red, green, and blue dimensions in reference to the range of intensity values used).

To further match low-level visual features, including spatial frequency spectrum and luminance histogram, the SHINE image processing toolbox^41^ was used with the software library extended to color images^38^. SHINE_color was used to match both the FFT spectrum and luminance histogram of the face and cat images over five iterations. For histogram matching, the option of optimizing the structural similarity (SSIM) was selected. For spectral matching, the whole amplitude spectra were equated for each orientation at each spatial frequency (*specMatch* option).

To balance variable (but slight) tilt in head orientation across pictures, mirror images were created yielding altogether 28 different stimuli. All stimuli subtended 6 visual degrees on a 27ʺ monitor and were presented on a gray background. Screen refresh rate was at 60 Hz. Stimulus duration was constantly 500 ms with a 1000-ms onset-to-onset interstimulus interval. Lights from the measurement booth were turned off while light emitted from the screen provided some ambient illumination.

During stimulus presentation, an animated rotation around the upright stimulus orientation was applied to increase attentional capture of the stimuli for infant participants. Sinusoidal angular rotation was defined by a magnitude of 10 degrees (5 degrees to left/right) and a rate of 2 Hz. To increase similarity between the adult and infant experiments, such animation was applied for adult viewers as well.

### Procedure

The experimental stimuli were presented to the participants in a passive recording condition, with no task besides attending to the stimuli. Adult participants were explicitly instructed to focus on the stimuli presented on the center of the screen. Infant participants sat on their caregiver’s lap in front of the computer screen used for stimulus presentation. Most infant participants’ attention was captured by the sequence of stimuli which was presented together with cheerful instrumental music played on the background. To maintain participants’ vigilance, background music was used also for adult participants. The content and phase of the background music was selected at random to cancel out any potential effects on visual ERPs.

**Figure 2:**
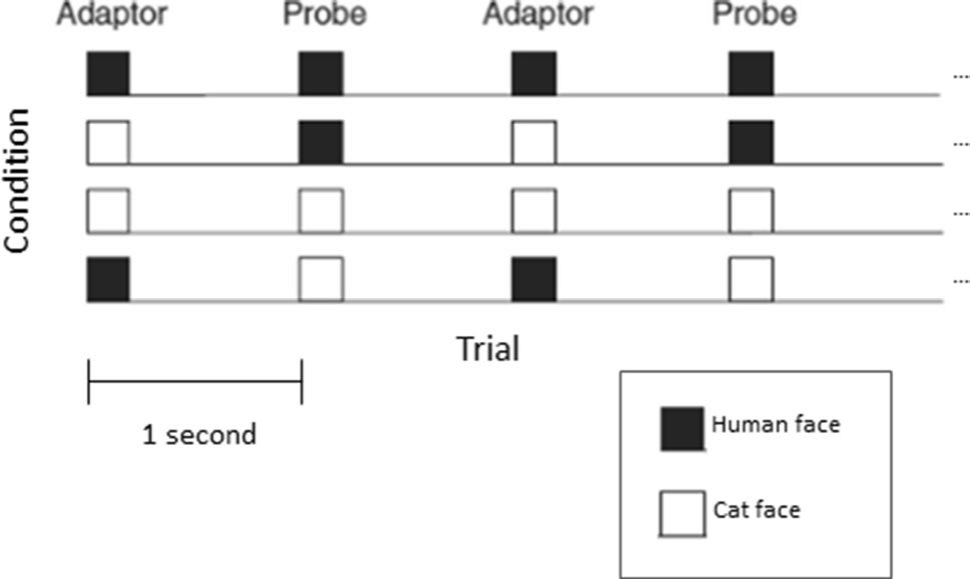
Schematic illustration of the stimulus-specific adaptation paradigm with two different stimulus categories of human (black squares) and cat (white squares) faces. ERPs were recorded in response to two different probe categories. Across separate stimulus conditions, the adaptor stimulus was chosen either from the same stimulus category as the probe or from the other stimulus category. In blocks with two different stimulus categories, human and cat faces alternated between consecutive trials.

Four different stimulus conditions were presented in the ERP experiment which were defined by the two stimulus categories (*F* = human faces, *C* = cat faces) being used as both probe and as adaptor stimuli (Fig. 2). These conditions included human face probe alternated with human adaptor (*FF*), human face probe with cat adaptor (*FC*), cat probe with cat adaptor (*CC*), and cat probe with human face adaptor (*CF*). These conditions were implemented by presenting three kinds of stimulus sequences in separate blocks of the experiment which consisted of either (1) human face stimuli (*FF*) alone, (2) cat stimuli (*CC*) alone, or (3) an alternation between human and cat faces (i.e., *FC*/*CF*). The stimulus exemplar for each trial was chosen randomly from the set of face pictures of the designated stimulus category. The conditions were counterbalanced across participants by varying the initial block type (1, 2, or 3) between participants.

Infant participants were presented with blocks consisting of 24 human or cat face images (one image per trial). Up to four iterations of each block were used for infant participants yielding from 24 to 96 trials per probe stimulus for each participant. For adult participants, each block consisted of 120 repetitions of each probe stimulus. The three different stimulus sequences (*FF*, *CC*, and *FC*/*CF*) were presented in six experimental blocks and up to 240 trials per probe stimulus. The duration of an experimental blocks (in s) was equal to the number of stimuli presented. The presentation of the stimuli was controlled with Psychtoolbox-3 library^42^ for MATLAB (MathWorks Inc., USA).

### EEG acquisition

EEG was recorded during stimulus presentation by using a high-density EGI HydroCel 128-electrode net (Electrical Geodesics, Inc., USA). Trigger signals synchronized with stimulus onset were sent online to the EEG acquisition system by the Psychtoolbox interface. Data were acquired until the predetermined number of blocks were completed or the infant participant could no longer remain attentive to the stimuli. A first order 0.1-Hz high-pass filter was used during the acquisition and data were sampled at 250 Hz. Electrode impedances were measured before the onset of the experiment.

### EEG analysis

#### EEG preprocessing

For infant participants, EEG preprocessing was based on a combination of video-based quality control (QC) and automated artifact detection using the Eegtool software^43^ which incorporates standard preprocessing functions from the EEGLAB toolbox^44^*.* Videos recorded during the EEG acquisition were inspected for participants’ excessive head and body movements, infant or parent touching the electrodes, parent moving the infant, fussiness, or participant losing interest towards stimuli on the scale of experimental blocks. Sequences of EEG data coincident with such artefact-inducing activities were rejected prior to EEG preprocessing and analysis.

Automated EEG preprocessing consisted of (1) low-pass filtering at 30 Hz, (2) segmenting epochs from − 100 to 500 ms with reference to stimulus onset, (3) initial baseline correction to epoch mean, (4) detrending the epoch, (5) rejecting channels with impedance values above 200 Ω, and (6) baseline-correction to the 100-ms pre-stimulus signal. The data in each epoch was then scanned (automatically) for artifactual EEG signal. Channels in each epoch indicating potentials greater than ± 150 µV were marked bad and were replaced with data interpolated from acceptable channels using spherical spline interpolation. However, if the number of bad channels in an epoch was greater than 12 (i.e., about 10% of the 128 electrode channels), the entire epoch was rejected. Finally, the EEG signal was re-referenced to the average from all electrodes.

For adult participants, similar preprocessing of the EEG signal with Eegtool was performed (no video QC needed). In addition, due to longer experiment duration and increased prevalence of blinks, ocular artefacts were removed from EEG data with Independent Component Analysis (ICA) using the EEGLAB toolbox. To increase the reliability of ICA, a preliminary preprocessing of the EEG signal was conducted prior to ICA with higher thresholds for excessive EEG values and number of bad channels (500 µV and 30 channels, respectively).

#### ERP extraction

**Figure 3:**
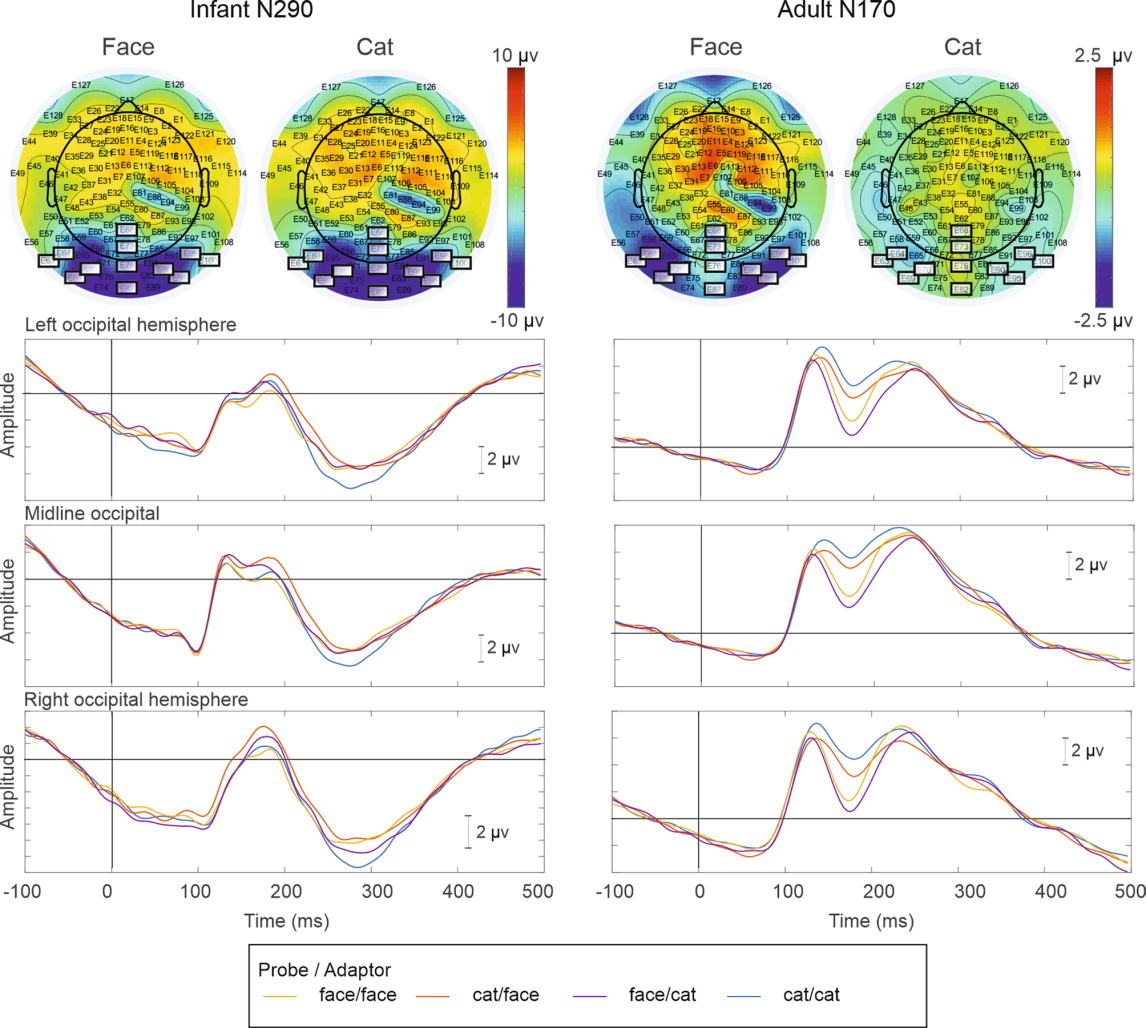
ERP responses elicited by human faces and cat faces in 7-month-old infants (left) and in adult (right) participants. Topoplots (top) represent scalp topography of infant N290 and adult N170 response as difference from the preceding P1 response. Lower panels show response waveforms that are averaged from electrode arrays located at left, midline, and right occipital regions (see main text for channel selection). Data from 49 infant, and 21 adult participants, respectively.

ERP waveforms were extracted by taking the algebraic average of EEG signal across epochs that were synchronized to the onset of the probe stimuli. Peak values of the ERP amplitude and latency within specific time intervals and initially from individual electrodes were extracted for further analysis. The time-windows for responses were chosen based on previous studies reporting P1, N170, and N290 responses, noting the age-specific differences. The grand-average waveforms (Fig. 3) were also visually inspected to verify the latency ranges for the ERP deflections in the current dataset. For adult participants, the N170 response was selected as the minimum value in the 148–192-ms interval. For infant participants the N290 was likewise extracted as the minimum value, but between 232 and 324 ms post-stimulus time. In addition, the P1 response preceding N170/N290 was extracted from 112–148 and 160–208-ms time ranges for adult and infant participants, respectively. Both peak amplitude of the response (maximum and minimum µV, for P1 and N170/N290, respectively) and the latency of the response were extracted. The N170/N290 amplitudes were inspected in reference to the prestimulus baseline. As recommended for developmental populations^22,45,46^, these were also extracted as peak-to-peak amplitudes from the preceding P1 maximum value.

The electrode arrays for ERP analysis were selected from the occipital regions indicating prominent N170/N290 responses in the grand average results. The N170/N290 response is typically recorded in the occipitotemporal electrode sites. The current electrode arrays were also similar or located in the vicinity of the electrodes yielding maximal difference in N290 response between face and non-face stimulus in our previous study with 5- and 7-month-old infants conducted with the same EEG equipment^11^. Three electrode locations were used for ERP extraction (P1, N170 and N290). These were the occipital left (E63, E64, E69, E70); midline (E68, E73, E82, E76); and right-hemispheric (E100, E96, E95, E90) sites. Arithmetic mean was calculated for ERP values across electrodes within each of the three channel sets.

In case of uniform stimulus sequences of human (*FF*) or cat (*CC*) faces, all stimuli were technically treated as “probe” so that all EEG segments marked by these triggers were included in the averaged ERP. In sequences of alternating human and cat stimuli, either human or cat stimulus was treated as the probe to which the averaged EEG segments were synchronized. Thus, from one EEG recording with alternating stimulus categories, two ERPs were averaged (*FC* and *CF*). The number of epochs averaged for infant ERPs across conditions *FF*, *FC*, *CC*, and *CF* was 43.0 (21.1), 45.9 (23.3), 45.0 (22.3), and 45.8 (22.9), respectively. For adult ERPs, the corresponding number of averaged epochs was 237.3 (2.9), 238.7 (2.7), 237.9 (2.0), and 238.6 (1.9). During EEG preprocessing on the average 8.0 (6.7) and 2.8 (2.4) additional epochs per condition were rejected due to artefacts for infant and adult participants, respectively.

In addition to the posterior activity attributed to the N170/N290, the ERP topography suggested concomitant parietal activity (Fig. 3, top). To sustain infants’ attention on the train of face stimuli, the faces were animated by angular rotation. As visual motion has been shown to elicit ERPs in the parietal regions of adults and infants (5 months old and younger^47^), it might be speculated that the currently observed parietal activity might be related to motion processing. Also face-sensitive activity has been previously recorded at parietal sites^48^. However, in the current study we focused on the activity recorded at more posterior electrode sites which were unaffected by the activity visible in the parietal channels.

For infant participants, we additionally calculated bootstrapped Standard Measurement Error (*bSME*) to evaluate signal quality of the N290 response. The mean *bSME* value for all N290 data included in the final analysis was 3.15 µV. This measurement error-based variability was smaller than the standard deviation 5.30 µV of the N290 amplitude values, suggesting that observed variability in N290 was mainly driven by individual and experimental factors rather than by measurement error^49^. The measurement error bSME varied by the electrode location [*F*(2, 45.70) = 10.86, *p* < 0.001; left = 3.37 uV; midline = 3.35 µV, and right = 3.01 µV)]. However, no differences in bSME values were found between stimulus condition [*F*(3, 33.26) = 0.37, *p* = 0.773] and there was no interaction between electrode site and stimulus condition [*F*(6, 32.49) = 1.89, *p* = 0.11].

### Statistical analyses

Linear mixed model (LMM) analyses were used to study repeated-measures effects of stimulus condition and electrode location on the amplitude and the latency of the P1 and N170/N290 responses. The factor of stimulus condition consisted of four levels, defined by combinations of two different probe (face and cat) and two different adaptor (face and cat) stimuli (i.e., *FF*, *CC*, *FC*, and *CF*). The factor of electrode location consisted of three levels corresponding to left-, mid-, and right-hemispheric sites. LMM was based on unstructured covariances. As dependent variables we used baseline-corrected and peak-to-peak amplitude of the P1 and the N170/N290 response as well as response latency. In the “Results” section, the amplitude values are presented as LMM estimated marginal means and as raw summary statistics in Table 1. All statistically significant LMM main and interaction effects, of stimulus condition, are reported in the “Results” section.

**Table 1:** Summary statistics of infant N290 and adult 170 amplitude and latency.

Probe	Adaptor	N290 amplitude (μV)		N290-P1 (μV)		N290 latency (ms)	
		Mean	*SD*	Mean	*SD*	Mean	*SD*
**Infant (7-month old)**							
Human	Human	− 8.42	5.30	− 10.02	4.91	278.2	23.5
Cat	Cat	− 9.31	6.08	− 11.79	5.60	277.8	22.7
Human	Cat	− 8.69	5.31	− 11.47	5.92	282.7	22.2
Cat	Human	− 7.90	4.29	− 11.06	5.40	283.6	22.8
All	All	− 8.59	5.30	− 11.11	5.50	280.6	22.9

Probe	Adaptor	N170 amplitude (μV)		N170-P1 (μV)		N170 latency (ms)	
		Mean	*SD*	Mean	*SD*	Mean	*SD*
**Adult**							
Human	Human	− 0.11	1.91	− 4.08	3.80	170.7	12.7
Cat	Cat	1.31	1.82	− 2.96	3.15	172.1	13.6
Human	Cat	− 0.65	2.28	− 4.58	4.04	172.0	12.7
Cat	Human	0.70	1.94	− 3.26	3.40	170.7	14.1
All	All	0.31	2.12	− 3.72	3.65	171.4	13.2

Amplitude values are indicated also as peak-to-peak values in which the amplitude of the P1 response is subtracted from that of the N290/N170.

Planned pair-wise comparisons in the amplitude and the latency of P1 and N170/N290 response measures were used to investigate (1) *sensitivity* to human faces as defined by the difference in cortical response to human vs. cat face probes, and (2) *specificity* to human (or cat) faces as defined by category-specific repetition effects in cortical response to human and cat stimuli. The effects of (1) probe category (human vs. cat) were investigated between conditions where each probe was preceded by an adaptor stimulus from the same category (*FF* vs. *CC*). The repetition effects (2) of adaptor category were defined as differences in cortical responses between conditions with a human face as paired with human vs. cat adaptors (*FF* vs. *FC*), and between conditions with a cat probe as paired with cat vs. human adaptors (*CC* vs. *CF*).

In analyses of infant P1 and N290, the threshold for minimum number of averaged epochs was set to 20 per stimulus condition. Response values from ERPs based on fewer than 20 epochs were rejected from further analyses. The final response measures for infants were then based on the average on 44.9 epochs (*SD* = 22.4) per stimulus condition. For adult participants, on the average 238.1 (*SD* = 2.5) epochs were averaged to extract ERP statistics from each stimulus condition.

Data from 49 and 21 for infants and adults, respectively, were entered into final analyses. These sample sizes were sufficiently powered to detect medium to small (d > 0.40, for infants) and large to medium (d > 0.66, for adults) sized differences between two dependent means at conventional alpha (0.05) and beta (0.80) levels.

## Results

### Adult ERP

#### P1 response

P1 was elicited at the average latency of 132 ms (*SD* = 10 ms), which was modulated by stimulus condition [*F*(3, 20) = 9.41, *p* < 0.001]. The amplitude of the P1 response for adult participants was above baseline value by 4.03(3.57) μV [*t*(20) = 5.17, *p* < 0.001, *d* = 1.16)]. Decreased latency was found for P1 elicited by human (131 ms, *SD* = 9 ms) as opposed to cat (*M* = 136 ms, *SD* = 8 ms) faces (*p* < 0.001, *d* = 1.02). No category-specific repetition effects were found in P1 latency in response to human (*p* = 0.864) or to cat face (*p* = 0.393) probes. No effect of stimulus [*F*(3, 20) = 2.32, *p* = 0.106] or interaction between stimulus and electrode location [*F*(3, 20) = 0.24, *p* = 0.977] was found for adult P1 amplitude.

#### N170 amplitude

Prominent N170 wave was found in adult participants in the 148–192-ms latency range (Fig. 3, right panel). Peak-to-peak amplitude of the adult N170 measured from the preceding positive-going P1 wave were between − 3.00 and − 4.60 μV across different stimulus conditions with a significantly negative-going amplitude [*M* = − 3.72, *SD* = 3.42; *t*(20) =  − 4.99; *d* =  − 1.11].

A main effect of stimulus condition was found on N170 amplitude [*F*(3, 20) = 18.48, *p* < 0.001]. The N170 wave was amplified by 1.43 μV (*SD* = 0.94 μV) in the human vs. cat condition (*p* < 0.001; *d* = 1.52; *FF* > *CC*). Category-specific release from adaptation was found for both human and cat probes. That is, the N170 amplitude increased by 0.54 μV (*SD* = 0.73 μV; *p* = 0.003, *d* = 0.74), when the human probe was alternated with cat adaptor in comparison to the condition where both probe and adaptor were human faces (*FC* > *FF*). An increase in N170 of 0.62 μV (*SD* = 0.69 μV, *p* = 0.001, d = 0.89), was also found when the cat probe alternated with human adaptor in comparison to the condition with a sequence of only cat stimuli (*CF* > *CC*).

In analyses of the peak-to-peak amplitude of the N170 from the P1 response, the above effect of stimulus condition was found as well [*F*(3, 20) = 9.33, *p* < 0.001]. Also, an interaction between stimulus and electrode site was found [*F*(3, 20) = 2.60, *p* = 0.040]. The peak-to-peak N170 amplitude was increased for human vs. cat condition by 1.11 (1.26) μV (*p* < 0.001; *d* = 0.88; *FF* > *CC*). The amplitude also increased by 0.50(0.64) μV for the N170 elicited by human face probes when paired with cat vs. human face adaptors (*p* < 0.002; *d* = 0.79), indicating category-specific repetition effects (*FC* > *FF*). However, the difference between N170 elicited by the cat probe between cat vs. human adaptors was not replicated in the peak-to-peak amplitude (*p* = 0.079, *d* = 0.40). While the above differences were found at left, midline, and right occipital sites (face vs. cat, and repetition effects for human face probe: *p*s < 0.01), the category-specific repetition effect for cat probes was reduced in the right-hemispheric sites (*p* = 0.327, *d* = 0.22).

#### N170 latency

The mean latency of the adult N170 was 171 ms (*SD* = 13 ms). No main effect of stimulus [*F*(3, 20) = 1.38, *p* = 0.279] or interaction between stimulus and electrode location [*F*(8, 20) = 2.36, *p* = 0.057] were found on N170 latency.

### Infant ERP

#### P1 response

The P1 response (Fig. 3, left) was elicited in infants at the average latency of 181 ms (*SD* = 11 ms). No effects of stimulus condition [*F*(3, 36.99) = 0.11, *p* = 0.952] or interaction between stimulus condition and electrode location were found [*F*(6, 34.40) = 2.11, *p* = 0.077] on infant P1 latency. The amplitude of the P1 response for infant participants was above baseline value by 2.33 (4.42) μV [*t*(48) = 3.68, *p* < 0.001, *d* = 0.53]. No main effect of stimulus condition [*F*(3, 36.91) = 1.36, *p* = 0.28] or interaction between stimulus and electrode location [*F*(6, 36.83) = 0.54, *p* = 0.77] was found on infant P1 amplitude.

#### N290 amplitude

A prominent N290 wave (Fig. 3, left) with amplitude significantly below the baseline level [*M* =  − 8.88 μV, *SD* = 4.11 μV, *t*(48) =  − 15.11, *p* < 0.001, *d* =  − 2.18] was found in the infant ERP data (Fig. 3, left). The amplitude of the N290 also varied by stimulus condition [*F*(3, 37.51) = 2.88, *p* = 0.049], being larger in the cat than in the human face condition by 1.70 μV (*p* = 0.025, *d* = 0.33; *CC* > *FF*). No category-specific release from adaptation of the N290 response was found in the condition with face probe. That is, no difference was found in N290 elicited by face probe across conditions with face (*M* =  − 8.19 μV, *SD* = 5.18 μV) vs. cat (*M* =  − 8.85 μV, SD = 4.83 μV) adaptor (*p* = 0.310). However, alternation of the cat probe with human adaptor yielded a decreased (*p* = 0.008, *d* =  − 0.40; *CF* < *CC*) rather than an increased N290 amplitude (− 8.04 μV, *SD* = 3.99 μV) when compared to the condition where both probe and adaptor stimuli were cats (− 9.89 μV, *SD* = 6.02 μV). No effects of electrode location [*F*(2, 44.94) = 1.50, *p* = 0.234] or electrode by condition interaction [*F*(6, 36.94) = 0.64, *p* = 0.696] were found on N290 amplitude.

In analyses of peak-to-peak amplitude (N290 vs. P1), an effect of stimulus condition was found [*F*(3, 35.10) = 2.96, *p* = 0.046]. Pairwise tests indicated increased amplitude for cat vs. face probe condition (where adaptor was of the same stimulus category as the probe) by 2.56 μV (*p* = 0.006, *d* = 0.42; *CC* > *FF*), which was consistent with the results found from baseline-corrected N290 peak amplitude. Alternation of cat probe with face vs. cat adaptor also yielded a decreased N290-P1 peak-to-peak amplitude (by 1.19 μV), but unlike for baseline-referenced N290, this effect was statistically not significant (*p* = 0.065, *d* = 0.27). Finally, category-specific repetition effect for the face probe condition was found on N290-P1 peak-to-peak amplitude by a 1.45-μV increase in amplitude for cat vs. face adaptor condition (*p* = 0.025, *d* = 0.34; *FC* > *FF*). The above effects (*CC* > *FF* and *FC* > *FF*) were found (p < 0.05) also after adding the number of averaged epochs as a covariate in the statistical model.

#### N290 latency

The latency of the infant N290 response centered around 281 ms (*SD* = 23 ms). No effects of stimulus condition [*F*(3, 36.19) = 2.13, *p* = 0.113] or interaction between stimulus condition and electrode location [*F*(6, 38.52) = 2.03, *p* = 0.084] were found on N290 latency.

## Discussion

The current study aimed to distinguish between models of the development of face-selective neural representations based on theoretical frameworks of cortical maturation and interactive specialization (IS). The key difference between the predictions of these domain-specific and domain-general models, respectively, relates to the category-specific tuning of the neural responses to faces across developmental and mature populations. Specific tuning across ages was predicted by maturational but not by the IS model^16,17^. Further, while both models are consistent with an overall sensitivity to faces, the IS framework especially emphasizes the importance of early biases or sensitivity as a prerequisite for subsequent developmental tuning of cortical regions to faces. We found cortical sensitivity to faces in adults as demonstrated by increased amplitude of the N170 response to human faces in comparison to the cat faces. In 7-month-old infants, the N290 response, a precursor of the N170 response^8^, was, in contrast, increased in amplitude in the cat condition relative to the human face condition. Stimulus-specific repetition effects, suggesting category-specific tuning in the underlying neural generators were found in both adult and infant participants.

Face-sensitive effects have been previously found in the P1 response, from 4 years to adulthood^45^ and in infants^8^. However, negative findings of the face-effect in infant P1 have been reported as well^46,50,51^ and, in adults, the response has been thought to reflect the influence of low-level visual cues^52^. In the current study, analysis of the P1 response was included, because larger P1 inevitably influences the amplitude and topography of the N170/N290 response. Further, the P1 response might serve as an index of the degree that the low-level visual cues have been matched across human and control face stimuli^52^. No face-related effects were found in the amplitude of the P1 response either in infant or adult participants.

In adults, analyses of N170 and P1-N170 peak-to-peak amplitude converged to indicate category-specific release from adaptation (or release from repetition suppression) for the response elicited by human face probes when alternated with cats rather than category-similar human faces. Such release from adaptation has been previously suggested to reflect the recruitment of distinct neural resources specific to the probe stimuli^21,25–27^, in relation to those responsive to the adaptor stimuli. Hence, the above repetition effects could be interpreted to indicate the activity of neural assemblies that are distinctly responsive to human faces in contrast to matched non-human faces. A release from adaptation was also found for the N170 elicited by cat probes paired with human adaptors, but the size of this effect was reduced in the P1-N170 peak-to-peak analyses. These results are consistent with previous ERP-based evidence of face-sensitive^12,53^ and face-specific^32–34,54^ processes underlying the N170 response and validate the current stimulus parameters for investigating these processes in infant participants.

In 7-month-old infants, we found differences in the N290 response amplitude that indicated variability in cortical activity elicited by the human vs. non-human face stimuli, as well as category-specific repetition effects. Contrary to that found in the adult N170 and in previous infant ERP studies^8–11^, the infant N290 was increased in amplitude in the non-human (cat) vs. human face condition. Moreover, the category-specific repetition effects for cat probes were found, but these were contrary to the predicted release from adaptation and to that found in the current results from adults. Namely, a decrease rather than an increase in N290 amplitude was found when the cat probes were paired with category-dissimilar human faces vs. category-similar cat faces. Finally, the results on infant N290 indicated category-specific release from adaptation for human face probes when these were paired with cat adaptors.

Evidence for specialized processing of facial cues has been previously indicated in categorical perception of facial expressions of emotion as well that have been found in the infant P400, elicited around 400 ms after stimulus, at the age of 7 months^55^. In the current study, categorization between human and cat faces was found at an earlier latency range of the N290. The current N290 results could indicate more rapid categorization of broad categories, human vs. non-human faces, in comparison to more intricate facial expression of emotion. The current experimental paradigm with relatively short inter-stimulus interval and stimulus duration were also optimized for the elicitation of the N290 response rather than longer latency P400 and Nc components also implicated in face processing in infants^8,9^.

In the current ERP waveforms, some drift in baseline period was observable, which raises the possibility of the contribution of some residual activity to the recorded ERPs^56,57^. Some residual activity from previous trials may have been introduced due to the use of constant, rather than jittered, ISI in recording event-related responses^58^. If this activity would deviate systematically between experimental conditions, it could have an influence on the amplitude values of the face-related N170 and N290 responses. As the N170/N290 was preceded by the P1 response, any residual projections would likely also shift the amplitude of P1 as well. Therefore, we corrected the N170 and N290 response amplitude with that of the P1 in additional analyses. Stimulus specific release from adaptation for human face probe (alternated with cats) was found for adults in both baseline- and P1-referenced amplitude and for infants in the P1-referenced condition. A similar analytic procedure has been endorsed by previous studies of infant face-related ERPs as well^29,45^. However, we cannot completely rule out the possibility of some residual activity that might influence the current ERP results despite the above corrections.

The increase in N290 amplitude for cat faces as opposed to human faces seems to contradict the concept of heightened cortical sensitivity to conspecific signals that has been suggested to emerge early on^1,59^. It is also contrary to the typical finding of increased N290 response to faces as opposed to different kinds of control stimuli typically reported in the infant ERP studies^8–11^. It is of note, however, that sensitivity of N170 and N290 to faces may depend on the characteristics of the control stimuli and the increased amplitude of these responses to human face as opposed to control stimuli has not been uniformly replicated in the literature. Previously Scott et al.^60^ have suggested, based on their results indicating invariance in N290 response across conditions with human vs. monkey faces in 9-month-old infants, that the underlying neural generators of this response are not tuned to pick up species-specific differences that are manifested in the adult N170 but rather reflect the structural encoding of the face configuration regardless of species.

In the current study, the eye region between the categories of human and cat face stimuli differed by larger size of the visible eye (i.e., sclera, iris, and pupil) in cat as opposed to human faces. The eye region has been suggested to be an especially influential feature in face processing as indicated by results from studies where the eyes or faces were experimentally manipulated (removed) while recording the N170 response^61–64^. As a similar effect is possible for the N290 as well, some of the current results might have been influenced by differences in specific features and especially the larger size (height) of cat eyes in comparison to human eyes. Especially, the finding of an increased N290 amplitude in infants in response to cat vs. human faces could be due to larger eyes in the former stimulus category. However, if the current results would primarily reflect activity of an eye detector, the alternation of human faces with large-eyed cats would presumably lead to increased suppression of human-elicited N170/N290 response. In contrast, we found the stimulus-specific release from adaptation in adult N170 and infant N290 response for human faces alternated with cat adaptors. Therefore, it seems unlikely that the category-specific responses to faces could be explained by smaller eye size in humans.

Another determinant of the N290 response are the repetition effects that alter the generation of this response across a stimulus sequence^21,65^. In addition to response suppression or adaptation with repeated probe presentation, a phenomenon known as repetition enhancement has been described. Repetition enhancement is defined as an effect opposite to repetition suppression or adaptation, where the magnitude of the response increases with increased repetitions of categorically similar stimuli. The direction of the repetition effect, that is, enhancement or suppression, has been suggested to depend on parameters such as novelty or familiarity of the stimuli and on the number of repetitions of the stimuli presented. In the current study, the cat faces used as control stimuli were arguably less familiar than human faces with estimated exposure to human faces being around 25% of waking time for young infants^66^. Repetition enhancement as opposed to suppression has been previously associated with increased encoding of novel stimuli^21^. While the number of stimuli presented to infant participants in the current study was well within the recommendations for infant ERP studies^67^, the feasible experiment duration in infant studies is much shorter than that used for adults. In line with previous literature^21^, such combination of relatively short stimulus sequence (duration < 1 min) with unfamiliar stimuli (cats), may have induced a heightened encoding of these stimuli leading to response enhancement.

The encoding account could also explain the repetition effects for cat probes: alternating the cat probes with human faces presumably interfered with the encoding process, which resulted in decreased amplitude of the N290. Thus, we tentatively argue that the increased N290 amplitude to cat vs. human faces and the observed decrease in N290 elicited by cat probes following human adaptor stimuli reflects a combination of encoding a novel stimulus category and interference in repetition enhancement, respectively. It is of note that there are also contradicting reports of increased infant N290 to familiar as opposed to unfamiliar faces^68,69^. However, a more recent study by Guy et al.^70^ failed to find increased N290 amplitude for familiar as opposed to unfamiliar stimuli, but instead found an N290 amplification with heightened attention, which is compatible with the encoding effect^21^ in the context of repetition enhancement.

Previous research on infant ERPs has suggested early cortical sensitivity to faces which has been indicated already at 3 months of age by an increased N290 amplitude to face vs. matched noise stimuli^10^ or to monkey faces^9^. Whether this sensitivity to faces is paralleled with category-specific processing of face configurations has received mixed findings. By categorical specificity to faces it is meant that the response is both invariable across featural changes within the category of face stimuli and that it is variable across changes that pertain to the essential features of face stimuli. Specificity of cortical activity to the configuration of faces has been investigated using the face inversion effect (FIE; inversion constitutes a change in essential configuration features of faces), which is indicated in the adult N170 by an increase in amplitude and latency for inverted as opposed to upright faces. Peykarjou et al.^46^ found FIE for faces but not for cars in 3-month-old infants, thus suggesting category-specific processing of faces. While FIE has been found by the age of 3 months in N290 response^46^, Halit et al.^9^, reported FIE in N290 and P400 at 12 months of age but not at 3 months. In the current study, categorical specificity to faces was investigated through stimulus-specific repetition effects which provide complimentary evidence to that found using the FIE. Our current results suggested that categorical representations of human faces are found by the age of 7 months. Furthermore, the current control stimuli for human faces, cats, had a roughly comparable overall configuration of eye and mouth regions. It might thus be argued that the categorization found in the current study reflects a relatively fine-grained detection of conspecific cues. Comparable evidence for specialized processing of facial cues has been previously indicated in categorical perception of facial expressions of emotion that have been found in the infant P400 at the age of 7 months^55^.

Categorical specificity of infant cortical activity in response to faces has been found previously using a rapid alternation paradigm where face stimuli are presented periodically and embedded in sequences together with other visual objects. This stimulation has been shown to induce or elicit a rhythmic EEG component that coincides with the frequency of face occurrence. Importantly, this response has been attributed to category-specific face processing in the cortical face areas in infants^20,22^. Categorization of faces was indicated by de Heering and Rossion^20^ against natural scenes in 4–6-month-old infants, and against monkey faces in 9-month-old infants by Peykarjou et al.^22^. Effect of categorization of human vs. ape faces in neurocognitive processes of 9-month-olds has also been studied with a repetition priming paradigm by Peykarjou et al.^29^. They found increased N290 amplitudes for human and ape probes when primed by human faces and attributed their finding to suggest categorization between human faces and non-human faces^29^. Evidence for specialized processing of facial cues has been previously indicated in categorical perception of facial expressions of emotion as well that have been found in the infant P400 at the age of 7 months^55^.

Peykarjou et al.^29^ used a repetition paradigm, where human and ape faces were presented consecutively in different permutations. They found that the N290 response amplitude and latency elicited by these stimuli were dependent on the changes across these (basic) level categories but not across changes between individual specimen within humans or ape categories. They thus suggested that the 9-month-old participants had formed category-specific neural responses of (at least) human faces that distinguished them from a resemblant other-species category. In this respect, their results are in line with our current results which suggest stimulus-specific categorization of human faces. Our current study differed from that of Peykarjou et al.^29^ as the age of participants was 9 months for Peykarjou in contrast to 7 months in the current study. According to Scherf and Scott^28^, there are developmental trajectories of different aspects of face processing (species, race, age, and gender biases). Individuation and specialization to conspecific faces has been shown to take place between 6 and 9 months^28^. Other research has indicated basic level categorization by 6–7 months of age to variable object categories, although at delayed latencies corresponding to the Nc response (ERP)^30^. The current results add to the literature by suggesting that categorical representation of human faces, that differentiate between human and other species faces, are formed by 7 months of age.

The currently used category-specific repetition paradigm is largely analogous with the above-mentioned methods in that the effects of preceding stimulus content on the neural response synchronized to a subsequent probe stimulus are investigated. Our findings also resemble those from the rapid alternation paradigm^22^ in that an increased response amplitude was found for face stimuli after a change in stimulus from one facial category to another (i.e., from cat to human faces). The priming paradigm by Peykarjou et al.^29^ differed from the current investigation with respect to stimulus material, stimulus duration and inter-stimulus intervals, making direct comparison between the results difficult. It may, however, be argued that both the current results and those of Peykarjou et al.^29^ converge on indicating face-specific categorization in the neural activity giving rise to infant N290 response at 7 and 9 months of age.

According to the framework of interactive specialization^16,71^, the infant brain is viewed as equipped with domain-general biases that are conducive for further development of domain-specific processes that emerge with experience and interaction with the environment. The IS account of the development of face processing has received support from fMRI evidence of progressive and regressive changes in the face-selectivity of multiple neuroanatomical regions also outside the fusiform gyrus^17^ which has been implicated as the predominant face-specific area in the adult brain^72^. This view has been proposed as an alternative to theories suggesting innate domain-specific accounts of specialized areas of function, such as detection and processing of conspecific faces. Consistent with the IS model, we initially predicted that the early biases to face stimuli would be manifested in increased response amplitude to human faces as opposed to control stimuli, indicating face-sensitivity. In addition, we predicted that specificity of the cortical responses to faces would be found in adults, but not in infants, through stimulus-specific repetition effects found between conditions with within-category versus between-category variability. To test this hypothesis, we presented empirical findings from one age group during which rapid development of face (and other social) processing has been found^55^. Contrary to the IS model, we found categorical specificity to faces in infant brain responses. Given the initial indication of feasibility for the current repetition paradigm in studying infant face processing, it could be possible to longitudinally track developmental trajectories in face processing with similar procedures in future studies.

Maturational, domain-specific, models of the development of face-selective brain regions, would, in contrast, suggest that electrocortical responses would simultaneously become sensitive and category-specific to human faces when compared to a control stimulus condition^14^. While sensitivity to faces, as indexed by an increased N290 to faces vs. cats was not found, we did find category-specific repetition effects. The current results indicated category-specific neural activity elicited by face stimuli, when alternated with cat stimuli, in the infant peak-to-peak N290 response that resembled that found in the adult N170. The current results, thus, suggest that at 7 months of age, human faces are categorically distinguished from other-species faces as found in the stimulus-specific repetition effects in the N290 response. In this sense, the current results are consistent with an already refined domain-specific processing of human faces during the second half of the first year of life.

Studies of the neural generators of the N290 response in infants during the first year of life have localized these to relatively confined regions of the lateral fusiform gyrus and immediately neighboring areas^8^ which suggest similar brain regions to be active in face processing across infants and adults^70,73^. Together with the current results this points to an early onset of specific tuning in highly localized brain regions as the basis for the development of face processing, which is consistent with the maturational model of development. While it could be possible that the category-specific processing of faces could also reflect the development of interactive specialization preceding the age of 7 months, this account seems to contrast the time scale of IS that has been previously described to continue into childhood and adolescence^14,17^. It is of note that the assertion of early domain-specific neural circuitry for face detection (or other social functions^74^) does not preclude further changes in or subsequent developmental plasticity of these neural mechanisms that would follow interactive specialization.

## Conclusions

We used a stimulus-specific repetition paradigm to study face-related cortical activity in adults and in 7-month-old infants. Sensitivity and categorical specificity of the adult N170 to human faces was found as predicted. In infants, the N290 response increased in amplitude for cat vs. human face stimuli, which might be attributable to encoding-related amplification of the N290 response to cat stimuli as a novel, unfamiliar, stimulus category^21^. To preclude confounds related to low-level visual features, careful control of color, luminance profile, and spatial frequency spectra was ensured by image manipulations and analyses. Category-specific repetition effects in the infant N290 (peak-to-peak) response were found as well, which resembled those found in adults showing release from adaptation for human faces when preceded by cat adaptors. Despite the need for caution in comparing the adult N170 and its infant precursors^2,9,10,75,76^, we suggest that the current face-specific repetition effects of the N290 indicate the formation of categorical representation of conspecific faces at 7 months of age with relatively narrow tuning properties that distinguish human faces from non-conspecific face stimuli. While aspects of face processing continue to develop through childhood and adolescence, the current results indicate early maturation of category-specific tuning, rather than a sensitivity bias, to human faces in the neural generators of the N290 response.

## Supplementary Information

Supplementary Information.

## Data Availability

The data will be made available anonymously at Zenodo (10.5281/zenodo.6801531).
