# Supplementary material for: Neural specialization to human faces at the age of 7 months
Source: Sci Rep. 2022 Jul 21;12:12471. doi: 10.1038/s41598-022-16691-5 (PMC9304373; doi:10.1038/s41598-022-16691-5)
Supplement: Supplementary file 1 — Supplementary Information. [file 41598_2022_16691_MOESM1_ESM.docx]

**Supplementary file for article:** Neural specialization to human faces at the age of 7 months

**Authors:** Yrttiaho, Santeri; Kylliäinen, Anneli; Parviainen, Tiina; Peltola, Mikko

Infant participants were recruited from Tampere and surrounding areas. Contact information for families with infants around 7 months of age during data collection (October 16th – December 7th, 2020) were drawn from the Finnish Digital and Population Data Services Agency. Out of 521 contacted families, 63 volunteered and 56 brought their infant child to a study visit. Data from altogether 49 (87.5%) infants [19 female, age = 30.5(0.5) weeks] were qualified into the final analyses. As background data we collected parent-reported temperament traits of infant participants using the IBQ-R short form (Putnam et al., 2014). The current participants were described by major temperamental factors of Surgency [M(SD) = 4.36(0.57)], Negative emotionality [M(SD) = 3.14(0.65)], and Regulatory capacity [(M(SD) = 4.94(0.58)]. No correlations between these traits and ERP measures were found. The IBQ-RS data will be made available anonymously at Zenodo (doi: 10.5281/zenodo.6801531).
